# Insights into the Therapeutic Use of *Kalanchoe pinnata* Supplement in Diabetes Mellitus

**DOI:** 10.3390/ph18101518

**Published:** 2025-10-10

**Authors:** Felix Omoruyi, Lauren Tatina, Lizette Rios, Dewayne Stennett, Jean Sparks

**Affiliations:** 1 Department of Health Sciences, Texas A&M University, Corpus Christi, TX 78412, USA; jean.sparks@tamucc.edu; 2 School of Osteopathic Medicine, University of the Incarnate Word, San Antonio, TX 78235, USA; lolo.12171998@gmail.com; 3 Department of Life Sciences, Texas A&M University, Corpus Christi, TX 78412, USA; lerios@UTMB.EDU; 4 The Transitional Year Programme, University of Toronto, Toronto, ON M5S 2E8, Canada; dewayne.stennett@utoronto.ca

**Keywords:** diabetes mellitus, *Kalanchoe pinnata*, antioxidants, inflammation, oxidative stress

## Abstract

*Kalanchoe pinnata*, commonly known as the “miracle plant” or “life plant”, is a succulent species traditionally used for various health conditions. Recent research investigations have intensified interest in this species due to its diverse repertoire of bioactive constituents, including flavonoids, alkaloids, triterpenes, and glycosides. These compounds have been associated with multiple therapeutic effects, notably antioxidant, anti-inflammatory, and antidiabetic activities. Although several studies have highlighted the positive effects of the extracts of *K. pinnata* on key factors contributing to the pathophysiology and complications of diabetes mellitus, a systematic overview focusing on the use of these extracts and their bioactive constituents in the management of the disease is lacking. This literature review summarizes the phytochemical composition, traditional uses, and recent scientific data supporting the antidiabetic potential of *K. pinnata*, with a particular focus on its effects on glycemic control, as well as inflammatory and oxidative homeostasis, toxicity, safety, and potential clinical implications. The phytochemical constituents discussed include quercetin, kaempferol, apigenin, epigallocatechin gallate (EGCG), avicularin, and bufadienolides, along with a presentation of representative structures. The review also covers the potential mechanisms of action in diabetes mellitus. The survey of available literature highlights the effects of *K. pinnata* on indices of diabetes mellitus, including enhancing insulin sensitivity, mitigating oxidative stress and inflammation, lowering blood glucose levels, and the potential adverse effects. These results point to the promising prospect for *K. pinnata* use in the management of diabetes mellitus and its associated complications, while underscoring the need for more rigorous investigations, including well-controlled clinical trials.

## 1. Introduction

According to the World Health Organization (WHO), diabetes mellitus is a chronic condition resulting from either insufficient insulin production or the body’s inability to utilize insulin effectively [1]. Insulin, produced by pancreatic beta cells, regulates blood glucose levels. Uncontrolled diabetes mellitus is marked by elevated blood sugar that can subsequently lead to complications such as neuropathy, nephropathy, retinopathy, and atherosclerosis. The International Diabetes Federation (IDF) estimated that 537 million adults had diabetes in 2021, with projections rising to 643 million by 2030 and 783 million by 2045 [2]. Diabetes mellitus encompasses three main types: Type 1 is caused by the autoimmune or environmental destruction of pancreatic beta cells, leading to absolute insulin deficiency. Type 2 diabetes is characterized by insulin resistance and impaired insulin secretion, resulting from beta-cell dysfunction. Gestational diabetes develops during pregnancy due to hormonal changes that impair insulin production, although blood sugar levels typically normalize after childbirth. The IDF also reported that diabetes caused 6.7 million deaths globally in 2021, with a total estimated health expenditure of 966 billion dollars [2]. As healthcare costs continue to rise, many are turning to alternative medicines for treatment and are combining it with modern medicine. Hence, medicinal plants now play a vital role in global healthcare, especially in the management of diabetes mellitus. Plants have long served as an essential source of therapeutic agents, with over 80% of current drugs derived directly or indirectly from them. Medicinal plants are often favored for their minimal side effects and have been used for centuries in traditional medical systems across many countries worldwide [3].

**Figure 1:**
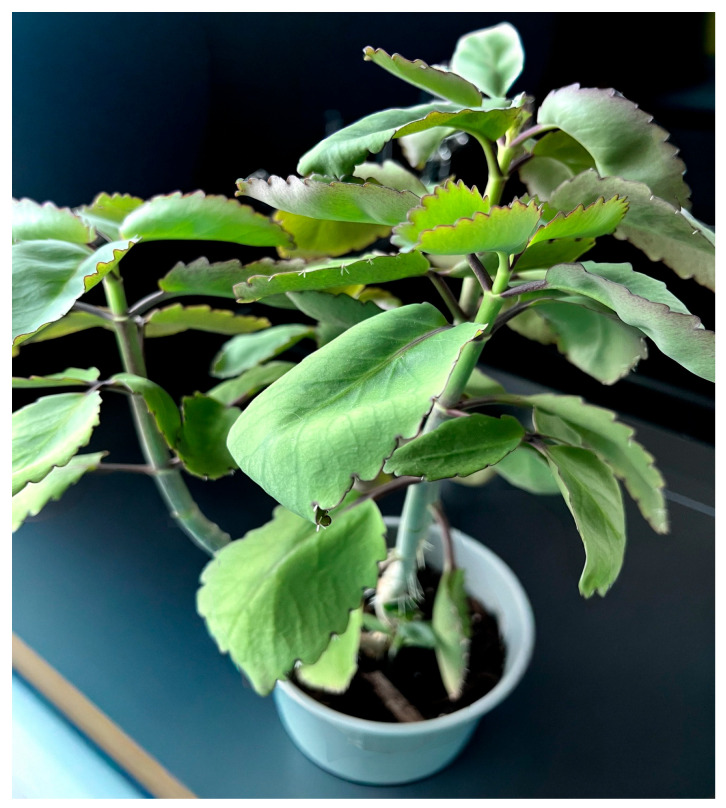
Image of *Kalanchoe pinnata*.

*K. pinnata* (Lam.) Pers., a member of the Crassulaceae family, is widely recognized for its medicinal uses across Africa, Asia, and South America. This perennial plant typically grows to a height of 1–1.5 m and features a moist, sturdy, tuberous, hairless stem. Its leaves are thick, simple, and lobed (3 to 5 lobes), while its drooping orange-red flowers are a distinctive feature. Notably, the plant produces vegetative buds—small reddish leaflets—along the leaf margins and stems, which can develop into new plants (Figure 1) [4]. *K. pinnata* is the most widely used species in its genus and has long been integral to traditional and holistic medicine worldwide [5]. It has been employed to treat a wide range of ailments, including wounds, cancer, urolithiasis, hypertension, diabetes, diarrhea, vomiting, respiratory and urinary tract infections, coughs, dysentery, migraines, and rheumatoid disorders [6,7,8,9]. Aqueous extracts from the leaves and roots are commonly used in traditional medicine for disease therapy in many regions, including the treatment of diabetes. The plant is known by various names—such as “miracle plant”, “air plant”, “life plant”, “master herb”, “cure-all”, and “best of luck leaves” reflecting its esteemed status in folk medicine [9,10].

Numerous studies have validated many of its traditional uses, revealing a broad range of therapeutic properties: analgesic, carminative, antiulcer, anti-inflammatory, antimicrobial, antioxidant, hepatoprotective, nephroprotective, cardioprotective, neuroprotective, tocolytic, antimutagenic, antitumor, antinociceptive, anti-edematogenic, immunosuppressive, diuretic, sedative, CNS depressant, muscle relaxant, and bone marrow modulatory effects [6,8,9,11,12]. Due to its potent antioxidant and anti-inflammatory properties, *K. pinnata* has drawn increasing interest in the context of chronic disease management. This review summarizes recent findings and prospects on the therapeutic potential of *K. pinnata* in managing diabetes mellitus.

## 2. *K. pinnata* Phytochemical Constituents’ Role in Diabetes Mellitus

**Figure 2:**
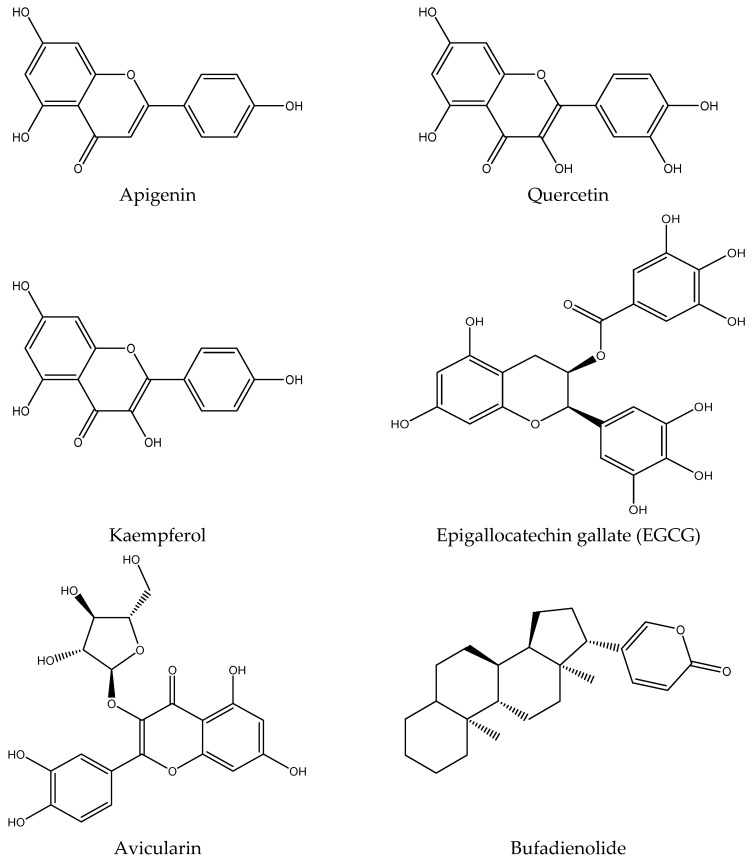
Structures of bioactive metabolites present in *K. pinnata* with potential antidiabetic activity.

Recent research has unveiled the remarkable phytochemical profile of *K. pinnata*, a plant renowned for its traditional medicinal uses. The phytochemical constituents include flavonoids, polyphenols, triterpenoids of ß-amyrin structure, phytosterols, alkaloids, glycosides, steroids, bufadienolides, lipids, and organic acids [13,14,15]. These bioactive molecules in the plant contribute to its antimicrobial and overall potential therapeutic properties [6,8]. The bioactive phytomolecules have been identified using Ultraperformance liquid chromatography fusion orbitrap mass spectrometry (UPLC-OT-FTMS) in an aqueous extract of *K. pinnata* leaves, locally grown in the Gulf Coast region of Corpus Christi, South Texas, USA [16]. The five main phytochemicals identified were quercetin, kaempferol, apigenin, epigallocatechin gallate (EGCG), and avicularin (Figure 2), each contributing to the plant’s antioxidant and therapeutic potential [16]. The compounds identified shared similarities in their chemical structures, though variations observed among the chemicals were primarily attributed to the presence and positioning of hydroxyl functional groups. These groups were either shifted from the phenol active group or were absent altogether. However, all compounds belonged to the same chemical class known as polyphenols, specifically flavonoids. Among the five main phytochemicals identified, three are flavonols, one is a flavone, and the remaining one is a catechin. Importantly, all compounds are recognized as bioactive molecules with documented antioxidant and anti-inflammatory properties, which may be due to the combined action of the bioflavonoids [16] (Figure 2, Table 1). The presence of various phytochemicals with various biological properties rationalizes the use of *K. pinnata* leaf extracts in folklore medicine [17]. Quercetin, a potent flavonoid found in *K. pinnata*, exhibits antidiabetic properties comparable to those of metformin, a widely used antidiabetic medication, in preclinical models [18]. It improves glycemic control, reduces lipid peroxidation, and protects β-cells by attenuating oxidative and inflammatory pathways [19]. By modulating key signaling pathways, quercetin shields cells from the damaging effects of reactive oxygen species (ROS) and promotes insulin production and release. Its ability to improve hyperglycemia makes it a compelling candidate for diabetes management. Similarly, EGCG, another key phytochemical in *K. pinnata*, has been shown to regulate blood sugar levels. Human trials further support its potential, revealing improvements in insulin sensitivity and metabolic health [20] and thus suggest that EGCG could be a valuable tool in the fight against type 2 diabetes. On the other hand, avicularin, a lesser known yet potent bioactive compound in *K. pinnata*, offers powerful antioxidant benefits. By modulating crucial cellular pathways such as phosphoinositide 3-kinase (PI3K), Akt/PKB, and MAPK [21], avicularin reduces oxidative stress and enhances cell survival. Studies highlight its ability to scavenge hydroxyl radicals, reducing inflammation-induced damage and potentially supporting cell growth [22,23]. Moreover, its positive impact on fasting blood glucose, lipid levels, and β-cell function further underscores its therapeutic promise for diabetic patients. Kaempferol, another flavonoid abundant in *K. pinnata*, has been recognized for its glucose-regulating abilities [24]. Kaempferol regulates lipid metabolism and improves insulin resistance. It also enhances insulin signaling and restores the balance between glucose utilization and production, thereby mitigating glucose toxicity [25]. Research indicates that kaempferol also enhances antioxidant activity by lowering malondialdehyde levels, a marker of oxidative damage [26]. These findings align with previous studies that confirm kaempferol’s role in combating oxidative stress and supporting metabolic health. Apigenin and kaempferol have been shown to influence inflammatory pathways. Notably, while both apigenin and kaempferol reduce TNF-α expression and secretion, apigenin uniquely decreases IL-10 levels, whereas kaempferol enhances IL-10 secretion [27]. This intricate interplay suggests that apigenin plays a nuanced role in immune modulation and inflammatory response, which could have implications for managing chronic diseases, including diabetes. Bufadienolides, on the other hand, influence apoptosis and autophagy [28].

## 3. Antidiabetic Potentials of *K. pinnata*

Diabetes mellitus is a group of metabolic disorders characterized by chronic hyperglycemia resulting from impaired insulin secretion, insulin action, or both. This condition leads to disturbances in the metabolism of carbohydrates, fats, and proteins, contributing to various metabolic dysfunctions. Millions of people around the world rely on medicinal plants to manage diabetes and other diseases, mainly due to the high cost of conventional treatments and their potential side effects. These plants are commonly used in developing countries, particularly among underprivileged populations, to treat many diseases, including diabetes. According to studies, 80% of people in underdeveloped nations rely on traditional therapies made from plants, which are the most frequently used type of treatment for various health conditions globally [29] (Table 2). Given the global prevalence of diabetes, over 800 medicinal plants have been identified as potential treatment options. However, the use of many of these plants for the therapy of diabetes and other diseases often lacks scientific validation. Critical information, such as proper dosages and potential side effects, remains unknown for many herbal preparations, making herbal medicine a potentially riskier alternative to modern treatments.

*K. pinnata* has gained attention in diabetes care due to its diverse medicinal properties, particularly its antioxidant, anti-inflammatory, and antihyperglycemic activities. The marked chronic hyperglycemia in diabetes induces oxidative stress and inflammation, contributing to complications such as neuropathy, nephropathy, and retinopathy. Hyperlipidemia has also attracted global attention due to its high prevalence and strong link to several life-threatening conditions, particularly cardiovascular diseases [36,37,38]. Studies suggest that *K. pinnata* exerts antihyperglycemic effects by modulating glucose metabolism, enhancing insulin sensitivity, and reducing oxidative stress in diabetic models. This makes it a potential candidate for managing diabetes-related complications. Singh & Pattnaik [30] (Table 2) also reported the antihyperlipidemic activity of the extract of *K. pinnata* leaves. Menon et al. [31] (Table 2) reported that the aqueous extract of *K. pinnata* may benefit diabetes management due to its ability to reduce body weight, lower blood glucose (hypoglycemic activity), and lower cholesterol levels. They noted that the decrease in blood glucose may be due to enhanced glycolysis in the liver, as evidenced by elevated pyruvate kinase activity in streptozotocin-induced diabetic rats. They reported an increase in blood urea nitrogen (BUN), which could raise concerns about potential renal damage due to *K. pinnata* supplementation. However, BUN is not the most sensitive marker of renal damage and may be elevated due to various unrelated factors. Additionally, the aqueous extract suppressed interleukin-6 (IL-6), a pro-inflammatory cytokine commonly elevated in diabetes. Goyal et al. [39] reported that administering the ethanolic extract of *K. pinnata* decreased blood glucose levels in diabetic rats. The plant extract also increased pancreatic insulin secretion. Matthew et al. [32] (Table 2) investigated the antidiabetic activity of ethanolic and aqueous extracts of the dried stem of *K. pinnata* in alloxan-induced diabetic rats. Both extracts demonstrated hypoglycemic activity. However, the ethanolic extract showed more inhibitory activity of the α-amylase enzyme than the aqueous extract, while the aqueous extract showed more hypoglycemic activity than the ethanolic extract. Another study revealed that the methanolic extract of the plant leaves exerted inhibitory action on α-amylase and α-glucosidase [29] (Table 2). The extract exhibited higher effectiveness in inhibiting these enzymes than standard acarbose [29,40]. Ojewole [33] (Table 2) also noted a remarkable decrease in the blood glucose levels in diabetic rats administered the aqueous extract of *K. pinnata*. The antidiabetic potential of *K. pinnata* leaves was investigated by Patil et al. [34] (Table 2) using streptozotocin-induced diabetic rats, focusing on the mechanisms underlying its antihyperglycemic effects and insulin secretion enhancement. The study examined four extract fractions: petroleum ether, chloroform, dichloromethane (DCM), and aqueous fractions. Among these, the DCM fraction demonstrated the most potent antihyperglycemic activity. Diabetic rats treated orally with 10 mg/kg body weight of the DCM fraction showed a notable reduction in fasting blood glucose levels, dropping from 228 mg/dL to 116 mg/dL. Glycated hemoglobin levels in this treatment group improved by 8.4% compared to the diabetic control group, which exhibited levels of 12.9%. Additionally, lipid profiles and insulin levels in the DCM-treated group closely resembled those of the non-diabetic control group. The DCM fraction of the plant demonstrated glucose-independent insulin secretagogue action, similar to that of the currently used drug glibenclamide, and therefore needs to be administered just before meals, as is the case with glibenclamide. Phytochemical analysis identified the active component of the DCM fraction as a phenyl alkyl ether derivative. These findings highlight the potential of the *K. pinnata* DCM fraction as a promising therapeutic option for diabetes mellitus, demonstrating its efficacy in promoting insulin secretion and improving glycemic control. Menon et al. [41] hypothesized that *K. pinnata* preparation consumption may act to increase peripheral insulin levels through extra-pancreatic insulin elevation and modulate the insulin–insulin receptor complex that may prolong the half-life of ligand-receptor complex that could sustain signaling similar to the report for AspB10 insulin analog [42]. The phytoconstituents are believed to act synergistically in regulating blood glucose, improving insulin dynamics, and combating oxidative stress, suggesting their potential use in managing diabetes mellitus. Overall, *K. pinnata* demonstrates multifactorial antidiabetic properties, making it a promising natural therapeutic candidate for diabetes management. While these findings are encouraging, more rigorous clinical trials are needed to validate *K. pinnata*’s efficacy and safety in human populations, establish optimal dosages, and develop standardized formulations for therapeutic use.

## 4. Effect of *K. pinnata* on Antioxidant Activities in Diabetes Mellitus

**Figure 3:**
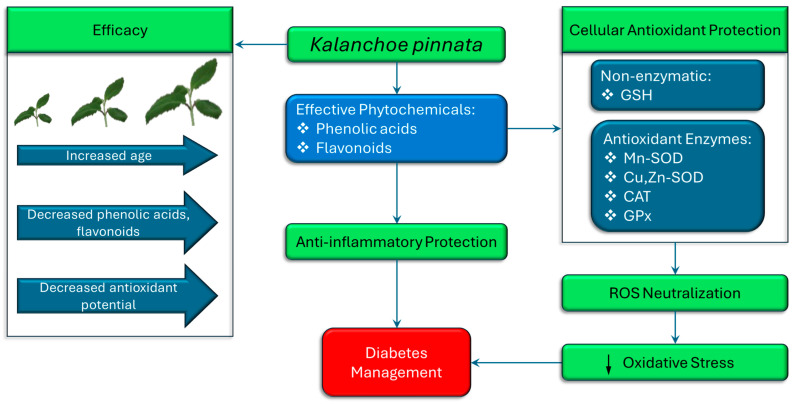
The therapeutic potential of *K. pinnata* in diabetes management, attributed to its antioxidant and anti-inflammatory properties. As the plant ages, levels of phenolic acids and flavonoids decline, resulting in a reduced antioxidant and anti-inflammatory potential and overall efficacy. Antioxidant protection involves both non-enzymatic (GSH) and enzymatic systems (Mn-SOD, Cu, Zn-SOD, CAT, GPx), which collectively neutralize reactive oxygen species (ROS) and reduce oxidative stress. These mechanisms ultimately support better diabetes management by mitigating oxidative damage and inflammation. (↓ denotes decrease).

Oxidative stress is a central contributor to the pathogenesis and progression of diabetes mellitus, particularly through its role in β-cell dysfunction, insulin resistance, and the development of diabetic complications. In this context, *K. pinnata* has garnered increasing scientific interest for its antioxidant properties, which may have therapeutic relevance in diabetes management (Figure 3). Studies have demonstrated that *K. pinnata* extracts possess robust free radical scavenging capacity, primarily attributed to their high content of flavonoids and phenolic acids. These phytochemicals act by neutralizing reactive oxygen species (ROS), thereby preventing oxidative damage to cellular components, including lipids, proteins, and nucleic acids. At the cellular level, antioxidant defense involves both enzymatic and non-enzymatic systems. Enzymes such as manganese superoxide dismutase (Mn-SOD/SOD2), copper-zinc superoxide dismutase (Cu, Zn-SOD/SOD1), catalase (CAT), and glutathione peroxidase (GPX) play pivotal roles in detoxifying ROS, alongside non-enzymatic antioxidants such as glutathione (GSH) (Figure 3), which help maintain redox homeostasis [43]. Ramon et al. [16] reported a remarkable increase in catalase activity following treatment with 400 μg/mL of *K. pinnata* extract in type 2 diabetic human skeletal muscle cells, suggesting its potential to mitigate oxidative damage in type 2 diabetes mellitus. This increase may reflect the synergistic action of multiple bioactive constituents, as also suggested by Uchegbu et al. [17]. Although catalase has a relatively low affinity for hydrogen peroxide (H_2_O_2_) compared to other peroxidases [44], its upregulation remains critical. Catalase-mediated detoxification of H_2_O_2_ becomes increasingly essential when peroxidase systems are saturated, thus protecting cells from cumulative oxidative damage. The anti-inflammatory and antioxidant activities of *K. pinnata* have mainly been linked to its natural flavonoid content. Pinheiro et al. [12] assessed the phytochemical profile of *K. pinnata* across different growth stages and geographic regions in Brazil. They observed that younger leaves exhibited higher levels of total phenolics and flavonoids, correlating with increased antioxidant potential. This developmental gradient suggests that phytochemical content, and thus therapeutic efficacy, can vary based on harvest time and environmental conditions, which is particularly relevant for standardizing formulations intended for use in diabetes management. Extraction methods and solvent choice further influence the yield and bioactivity of *K. pinnata* extracts. Saeed et al. [45] demonstrated that supercritical fluid extraction at 5500 psi and 40 °C for 2 h yielded extracts with high concentrations of phenolics and flavonoids, thereby enhancing their antioxidant capacity. Selecting appropriate solvents based on polarity and volatility is essential for preserving these compounds during processing [46]. Collectively, these findings support the potential application of *K. pinnata* as a natural antioxidant agent in the management of diabetes. By modulating oxidative stress pathways and supporting endogenous antioxidant defense mechanisms, *K. pinnata* may provide a complementary strategy to enhance glycemic control and mitigate the risk of oxidative damage associated with chronic hyperglycemia.

## 5. Anti-Inflammatory Potentials of *K. pinnata* in Diabetes Mellitus Management

**Figure 4:**
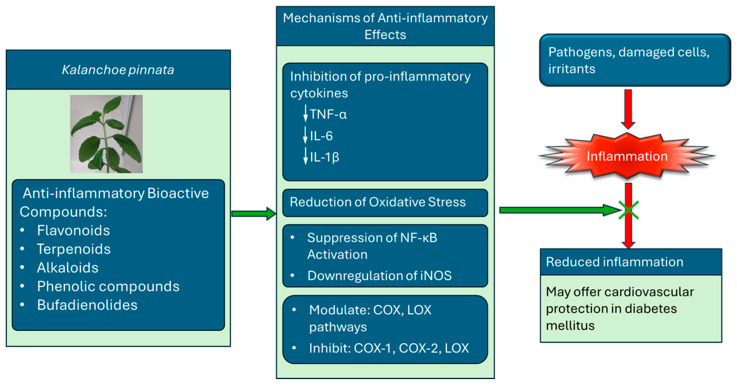
The anti-inflammatory mechanisms of bioactive compounds in *K. pinnata* and its potential cardiovascular benefits in diabetes mellitus. The plant exerts its effects by inhibiting pro-inflammatory cytokines (TNF-α, IL-6, IL-1β), reducing oxidative stress, suppressing NF-κB activation, and downregulating iNOS expression. The compounds also modulate cyclooxygenase (COX) and lipoxygenase (LOX) pathways, inhibiting COX-1, COX-2, and LOX enzymes. Collectively, these actions help block inflammation and improve cardiovascular outcomes in individuals with diabetes (↓ denotes decrease).

Inflammation is a complex biological response to harmful stimuli, including pathogens, damaged cells, and irritants. Chronic inflammation is associated with various diseases, including arthritis, diabetes, and cardiovascular disorders. Natural compounds from medicinal plants have gained attention for their potential role in modulating inflammatory pathways. *Kalanchoe pinnata* has been traditionally used to treat inflammation-related conditions. The bioactive compounds responsible for the anti-inflammatory activity of *K. pinnata* include flavonoids, terpenoids, alkaloids, phenolic compounds, and bufadienolides. These secondary metabolites exhibit potent anti-inflammatory effects by inhibiting pro-inflammatory mediators and oxidative stress [47]. The anti-inflammatory effects of *K. pinnata* are attributed to multiple mechanisms, including the inhibition of pro-inflammatory cytokines (Figure 4). Studies indicate that *K. pinnata* extracts reduce levels of key cytokines such as TNF-α, IL-1β [26,48], and IL-6 [34,35] (Table 2), which are involved in inflammatory signaling. The plant extract has also been found to modulate the cyclooxygenase (COX) and lipoxygenase (LOX) pathways and inhibit cyclooxygenase-1 (COX-1) and cyclooxygenase-2 (COX-2) [11,49] and LOX enzymes, reducing the synthesis of inflammatory prostaglandins and leukotrienes. The flavonoids and phenolic compounds also contribute to reducing oxidative stress, a notable factor in chronic inflammation. *K. pinnata* has been reported to suppress the activation of NF-κB, a transcription factor that regulates the expression of pro-inflammatory genes. The extracts also downregulate inducible nitric oxide synthase (iNOS), limiting excessive NO production and contributing to inflammatory damage. Aging and diabetes are associated with elevated systemic levels of IL-1β, TNF-α, and IL-6 [50]. Pro-inflammatory cytokines, such as TNF-α and IL-1β, contribute to atherosclerosis by inducing the expression of E-selectin, intercellular adhesion molecule 1 (ICAM-1), and vascular cell adhesion molecule 1 (VCAM-1) in endothelial cells [51]. Kong et al. [52] identified IL-1β and TNF-α as key mediators in the initiation and progression of atherosclerosis. Conversely, Menon et al. [34] (Table 2) reported elevated IL-1β and TNF-α levels in diabetic rats administered an aqueous *K. pinnata* preparation, indicating that it may not effectively mitigate the risk of atherosclerosis.

However, the aqueous preparation notably reduced the levels of IL-6, a cytokine upregulated in diabetes and known to trigger endothelial dysfunction, which can lead to atherosclerosis [53]. The hypothesis is that IL-6 downregulation may counteract the adverse effects of IL-1β and TNF-α, suggesting that *K. pinnata* may offer cardiovascular protection. Overall, *K. pinnata*’s remarkable anti-inflammatory properties are attributable to its diverse range of bioactive compounds, such as flavonoids, steroids, triterpenoids, and phenolic compounds. Its notable anti-inflammatory activity involves multiple biochemical pathways, including the suppression of cytokines, inhibition of enzymes, and antioxidant effects (Figure 4). The phytochemical profile supports its potential development as a plant-based therapeutic agent.

## 6. Potential Mechanism of *K. pinnata* Therapy in Diabetes Mellitus

**Figure 5:**
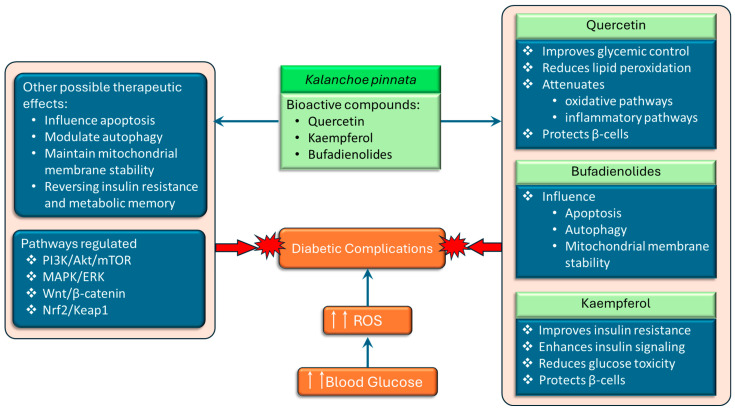
The schematic representation of the potential therapeutic effects of *Kalanchoe pinnata* bioactive compounds, quercetin, kaempferol, and bufadienolides, which exert protective effects by targeting multiple mechanisms. Quercetin improves glycemic control, suppresses oxidative and inflammatory pathways, and protects pancreatic β-cells. Kaempferol enhances insulin signaling, reduces glucose toxicity, and protects β-cells. Bufadienolides influence apoptosis, autophagy, and mitochondrial membrane stability. Collectively, these compounds modulate key signaling pathways (PI3K/Akt/mTOR, MAPK/ERK, Wnt/β-catenin, and Nrf2/Keap1) and may offer additional therapeutic benefits, including the reversal of insulin resistance, modulation of metabolic memory, and stabilization of mitochondrial function (↑↑ denotes increase).

*Kalanchoe pinnata* extracts have been widely reported to exhibit potent antioxidant and anti-inflammatory properties [26,47], which are particularly relevant in the context of diabetes mellitus, where oxidative stress and chronic low-grade inflammation play central roles in the pathogenesis and progression of the disease. In diabetes, elevated blood glucose levels lead to the overproduction of reactive oxygen species (ROS), which impairs insulin signaling, damages pancreatic β-cells, and contributes to the development of vascular complications. The plant’s flavonoid-rich extracts have been shown to regulate redox-sensitive signaling pathways implicated in both cancer and metabolic disorders (Figure 5). One of the central mechanisms by which *K. pinnata* may exert therapeutic effects in diabetes is by modulating intracellular reactive oxygen species (ROS) levels, restoring mitochondrial function, and enhancing the activity of antioxidant defense systems.

In diabetes, these mechanisms are critical for maintaining β-cell integrity and regulating programmed cell death in insulin-resistant tissues. The modulation of autophagy also holds therapeutic potential, as impaired autophagic flux contributes to β-cell failure and insulin resistance. Importantly, *K. pinnata* and its bioactive compounds regulate multiple cellular signaling pathways that are also dysregulated in diabetes (Figure 5), including the phosphoinositide 3-kinase/protein kinase B/mammalian target of rapamycin (PI3K/Akt/mTOR), mitogen-activated protein kinase/extracellular signal-regulated kinase (MAPK/ERK), wingless/integrated signaling/β-catenin (Wnt/β-catenin), and nuclear factor erythroid 2–related factor 2/kelch-like ECH-associated protein 1 (Nrf2/Keap1) pathways [54]. The PI3K/Akt/mTOR axis plays a pivotal role in insulin signaling, glucose uptake, and lipid metabolism. The activation of this pathway is impaired by insulin resistance resulting in a worsening of insulin resistance and decreased glucose uptake, ultimately leading to hyperglycemia. The MAPK/ERK pathway is involved in cell growth, differentiation and stress response and contributes to disease progression when overactivated in type 2 diabetes by promoting inflammation, insulin resistance and other diabetic complications. Wnt/β-catenin signaling is involved in insulin secretion, β-cell proliferation, and glucose metabolism, and dysregulation of this pathway contributes to the development of type 2 diabetes and its complications. The Nrf2 pathway is a master regulator of antioxidant gene expression. Activation of Nrf2 can restore redox balance and protect against diabetic complications, particularly nephropathy, retinopathy, and cardiomyopathy. Additionally, flavonoids like quercetin and kaempferol may act as epigenetic modulators, potentially reversing insulin resistance and metabolic memory by regulating DNA methylation, histone modification, and non-coding RNAs [54]. This offers a promising avenue for addressing the long-term effects of hyperglycemia even after glucose levels are normalized. These pathways are all potential targets for *K. pinnata* phytochemicals. Taken together, the antioxidant, anti-inflammatory, and signaling-regulatory activities of *K. pinnata* and its key constituents support its potential as an adjunctive therapeutic agent in diabetes management. By mitigating oxidative stress, modulating metabolic signaling pathways, and protecting insulin-producing cells, *K. pinnata* may help reduce the progression of diabetes and its associated complications.

Oufir et al. [55] identified four major bufadienolide components in *K. pinnata* grown in Brazil and Germany: bersaldegenin-1-acetate (1), bersaldegenin-3-acetate (2), bryophyllin A (3), and bersaldegenin-1,3,5-orthoacetate (4). In contrast, Stefanowicz-Hajduk et al. [56] found that bersaldegenin-2-acetate and bersaldegenin-5-acetate were the predominant bufadienolides, along with six additional bufadienolide compounds, in ethanolic extracts of *K. pinnata* cultivated in Poland. These findings suggest that the occurrence and composition of bufadienolides in *K. pinnata* vary with geographical origin. The bufadienolides present in *K. pinnata*, which have been previously studied for their anticancer effects, have been shown to influence apoptosis, autophagy, and mitochondrial membrane stability [28].

## 7. Organ Protection Properties of *K. pinnata* in Diabetes Mellitus

Organ function is critically important in diabetes management, as chronic hyperglycemia and subsequent oxidative stress can impair multiple organs, including the liver, kidneys, and lungs. Complications such as diabetic nephropathy, non-alcoholic fatty liver disease, and increased susceptibility to infections are well-documented in diabetic individuals. In this context, *K. pinnata* has demonstrated promising protective effects on organ systems implicated in diabetes-related pathology. Several in vivo and in vitro studies have highlighted the hepatoprotective and nephroprotective properties of *K. pinnata* leaf concentrate and ethanolic extract. Yadav and Dixit [57] reported that the administration of *K. pinnata* leaf concentrate remarkably lowered serum bilirubin levels in experimental models, indicating potential in supporting liver detoxification and jaundice management. Moreover, both the leaf concentrates and ethanolic extract reduced thiopental-induced sleep duration in rats exposed to carbon tetrachloride (CCl_4_), with histological findings showing that the ethanolic extract more effectively preserved hepatocyte integrity. These hepatoprotective effects are particularly relevant in diabetes, where oxidative stress can accelerate liver injury and metabolic dysregulation. In terms of kidney function, which is commonly compromised in diabetes due to diabetic nephropathy, *K. pinnata* has shown nephroprotective effects. Harlalka et al. [7] demonstrated that an aqueous extract of *K. pinnata* notably reduced blood urea levels and preserved serum and urine creatinine levels in gentamicin-induced nephrotoxicity in rats. The extract also enhanced urine output and maintained body weight, while its antioxidant properties outperformed ascorbic acid in free radical scavenging and inhibition of lipid peroxidation, mechanisms known to be beneficial in slowing the progression of renal damage in diabetes. Further supporting its nephroprotective role, Bigoniya et al. [58] found that *K. pinnata* improved kidney function and reduced renal crystal deposition in a model of sodium glyoxylate-induced nephrolithiasis. These improvements included increased urine volume and decreased excretion of oxalate, citrate, phosphate, and calcium, parameters often dysregulated in individuals with diabetic kidney stones. Histological analysis confirmed reduced tubular damage and inflammation. Emerging nanotechnology-based research also suggests novel applications of *K. pinnata* in diabetes-related urolithiasis. Priya et al. [59] synthesized silver nanoparticles using *K. pinnata* leaf extract and demonstrated dose-dependent inhibition of calcium hydrogen phosphate dihydrate crystal formation in vitro, with up to 84.8% inhibition at a 5% concentration. Similarly, Ranaweera et al. [60] found that *K. pinnata* extract slowed calcium oxalate stone precipitation, enhanced dissolution, and reduced crystal size and density. These findings are significant, as diabetic patients are at higher risk of nephrolithiasis due to altered urinary composition and oxidative stress. Moreover, while the respiratory system is not a classical target of diabetic complications, recent studies suggest an increased risk of pulmonary infections and inflammation in diabetic patients. In this context, *K. pinnata* may offer some protective benefits. Ehi-Omosun & Etunim [61] used bifenthrin to induce interstitial pneumonitis in rats and observed that a 200 mg/kg dose of *K. pinnata* leaf extract effectively mitigated pulmonary tissue damage. Although the higher dose (40,000 mg/kg) offered partial protection, its efficacy was comparatively reduced. These findings suggest a dose-dependent pulmonary protective effect, which may be relevant for preserving respiratory health in diabetic individuals. The organ-protective effects of *K. pinnata*, particularly on the liver and kidneys, are likely mediated by its high content of flavonoids, polyphenols, and organic acids, which exert strong antioxidant and anti-inflammatory actions. Given that diabetes-related complications are largely driven by oxidative stress and metabolic dysregulation, the use of *K. pinnata* as a natural adjunct therapy in diabetes care warrants further exploration. However, extreme caution and supervision must be employed when administering *K. pinnata* as a therapeutic agent, as chronic exposure to its leaf extract can lead to the downregulation of genes essential for proper organ function and defense against infections [62].

## 8. Toxicity and Safety of *K. pinnata*

*Kalanchoe pinnata* and other *Kalanchoe* species are important herbs in the traditional medicine of Asia and Africa, commonly used for their reported anti-inflammatory, antimicrobial, antidiabetic, and wound-healing properties. Despite this therapeutic potential, safety and toxicity concerns have been raised, mainly due to the presence of bufadienolides. Oufir et al. [55] quantified bufadienolides in *K. pinnata* leaves grown in Brazil and reported total concentrations ranging from 16.28 to 40.50 mg/100 g dry weight, compared with lower levels of 3.78 to 12.49 mg/100 g dry weight in those grown in Germany. Kolodziejczyk-Czepas and Stochmal [63] highlighted significant gaps in the literature regarding the distribution of bufadienolides across *Kalanchoe* species and their specific plant organs. Toxicological studies have shown mixed findings. Saravanan et al. [64] reported that the median lethal dose (LD_50_) of ethanolic leaf extract of *K. pinnata* was greater than 2000 mg/kg body weight, indicating low toxicity in rats. Repeated-dose studies revealed no adverse clinical signs, with normal hematological, biochemical, and histopathological results. However, Bhavsar and Chandel [65] reported that fresh leaf juice caused dose-dependent changes in cultured human blood lymphocytes. While 50 mL of juice produced only slight, non-significant increases in sister chromatid exchanges (SCEs)/cell and SCEs/chromosome, the 70 mL led to significant changes in the mitotic index (MI), SCEs/cell, and SCEs/chromosome. The cell cycle proliferative index, average generation time, and population doubling time values were non-significant for both doses when compared to the controls. They concluded that although the juice can be used pharmaceutically and traditionally, higher doses or prolonged use may induce genotoxic and cytotoxic effects. Chronic exposure of zebrafish embryos to *K. pinnata* per leaf (KPL) extract impaired development and altered behavior in a dose-dependent manner, significantly downregulating genes associated with multiple pathways [62]. The formulation was proposed to cause proteasome degradation, DNA damage, cell cycle arrest, apoptosis, and endocrine dysfunction—potentially leading to developmental delay. Interestingly, it also reduced anxiety-like behaviors, possibly through anti-inflammatory and anti-apoptotic mechanisms that may counteract proteasome inhibition. However, Martins Fernandes Pereira et al. [62] cautioned against the indiscriminate use of KPL extracts during pregnancy, emphasizing the need for further studies on their mechanisms of action and safe dosage. Evidence suggests that the extract may disrupt the endocrine system by inhibiting progesterone-mediated oocyte maturation and interfering with the insulin signaling pathway, potentially leading to mild inflammation, insulin resistance, and obesity [66]. Additionally, KPL may induce DNA damage by impairing replication and repair processes, leading to genetic alterations. If these mutations affect genes that regulate cell growth, they could contribute to cancer development [67]. Bufadienolides themselves are associated with cardiotoxicity. They have been shown to induce atrioventricular block, ventricular arrhythmia, and other cardiac dysfunctions [68,69]. Lin et al. [70] reported that resibufogenin, a bufadienolide, caused arrhythmias and myocardial injury, likely due to its reactive epoxy ring structure, which inhibits the sodium–potassium pump (Na^+^/K^+^-ATPase) and disrupts ionic homeostasis [71,72,73]. However, the coexistence of multiple components in traditional Chinese medicine may modify these effects. Synergistic interactions among bufadienolides have been proposed to enhance pharmacological activity, such as anticancer effects against prostate cancer cells, while maintaining a more favorable safety profile [74]. Although *K. pinnata* extract demonstrates a generally favorable safety profile at traditional and experimental therapeutic doses, it may carry potential toxicity risks due to bufadienolides, particularly concerning cardiotoxicity. Hence, the lack of clinical evidence considerably limits the therapeutic use of *K. pinnata*.

## 9. Potential Clinical Implications of *K. pinnata* Therapy

The collective findings from existing studies highlight the therapeutic potential of *K. pinnata*, particularly its role in preclinical studies in diabetes management. Traditionally used in various medical systems for treating a wide array of conditions, *K. pinnata* has demonstrated protective effects on organs commonly affected by diabetes, including the liver, kidneys, cardiovascular system, and pancreas. The potential clinical applications of *K. pinnata* stem from its rich content of bioactive compounds, including flavonoids, triterpenoids, steroids, bufadienolides, and phenolic acids, which contribute to its diverse therapeutic effects. Preclinical studies have demonstrated that *K. pinnata* can lower fasting blood glucose levels, provide antioxidant protection to pancreatic cells, and exhibit metabolic-regulatory activities and remarkable anti-inflammatory activity, which is believed to be mediated by flavonoids like quercetin. Quercetin inhibits pro-inflammatory enzymes and cytokines. In diabetes care, *K. pinnata* offers a compelling complementary approach. Its ability to modulate oxidative stress, support insulin signaling pathways, and protect against diabetic complications such as nephropathy, hepatopathy, and endothelial dysfunction suggests it may enhance conventional treatment regimens. In some studies, extracts of *K. pinnata* have shown comparable efficacy to standard therapies, often with fewer side effects and potentially lower costs. However, despite promising preclinical data, notable gaps remain in our understanding of *K. pinnata*’s mechanisms of action, safety profile, dose–response relationships, and long-term effects—particularly in the context of diabetes. For example, a recent report by Martins Fernandes Pereira et al. [62] documented gene downregulation in zebrafish following chronic exposure to *K. pinnata*. This finding may have potential health implications in humans. Further research is urgently needed to validate these findings in rigorous clinical trials, explore the effects across different age groups and comorbid conditions, and determine optimal formulations and dosing for therapeutic use. Many patients use *K. pinnata* and other supplements without informing their healthcare providers, thereby increasing the risk of potential drug–supplement interactions. From a clinical perspective, healthcare providers should initiate open conversations with patients about the use of herbal supplements, including *K. pinnata* [75]. To prevent potential adverse outcomes, clinicians should proactively inquire about supplement use and thoroughly assess the patient’s overall clinical status, including current medications, metabolic function, and organ health. Additionally, the quality, sourcing, and preparation of *K. pinnata* products are of paramount importance. Adverse side effects associated with herbal supplements often arise not from the herb itself, but from contamination, adulteration, or incorrect labeling. Patients should be encouraged to select products certified by reputable third-party organizations that adhere to Good Manufacturing Practices (GMPs), such as the United States Pharmacopeia (USP), the National Sanitation Foundation (NSF) International, or ConsumerLab. Notably, however, the differences between the doses used in clinical trials and those used in traditional remedies must be considered, along with their varying effects on different age groups. Regulatory resources, such as the NIH’s Dietary Supplement Label Database and the FDA’s MedWatch program, can also help clinicians and patients identify reliable products and report adverse events. The U.S. National Institutes of Health (NIH) offers several resources describing various *K. pinnata* products currently on the market for researchers and consumers alike. Its Dietary Supplement Label Database includes listings of available *K. pinnata* supplements. For example, Hawaii Pharm provides numerous supplements containing four fl. oz. of plant extract. The NIH National Library of Medicine also offers reliable information about herbal supplements. One such example is RelieveIt@ Regenerating Gel, which contains *K. pinnata* and other medicinal herbs as active ingredients. These sites are valuable resources for healthcare providers when recommending herbal supplements for their patients. Importantly, different species within the *Kalanchoe* genus exhibit remarkable variations in their chemical compositions and should not be used interchangeably. The standardization and identification of bioactive components are critical for ensuring safety and efficacy in diabetes care.

Overall, *K. pinnata* supplementation shows promising therapeutic potential in the management of diabetes. While its traditional use is widespread and some preclinical studies provide supportive evidence, more rigorous studies, including well-controlled human clinical trials, standardization of extracts, and toxicological profiling, are needed to establish its safety, optimal dosage, effects across different age groups and comorbid conditions, and long-term benefits. This literature review is intended for research purposes only and does not recommend therapeutic use without clinical validation. The promising therapeutic potential of *K. pinnata* is primarily based on in vitro and animal studies, which provide only indirect evidence for its safety and effectiveness in humans.

## 10. Conclusions

*Kalanchoe pinnata* may hold considerable promise as a natural adjunctive therapy in the management of diabetes mellitus. With continued research, it may contribute meaningfully to integrative therapeutic strategies aimed at improving metabolic control, reducing oxidative and inflammatory damage, and protecting against the development of diabetic complications. As interest in plant-based treatments grows, a thoughtful, evidence-based approach to their use, paired with stringent regulation and patient education, will be crucial to ensure the safe and effective incorporation of these treatments into diabetes care. However, despite the traditional use of *K. pinnata* for diabetes management, clinical trials are lacking, resulting in its current use being mostly complementary.

## Figures and Tables

**Table 1:** Shows the biological activities of several phytochemicals from *K. pinnata* that suggest antidiabetic potential.

Reference(s)	Bioactive Compound	Mechanism of Antidiabetic Activity
Ozsoy et al., 2020 [19]	Quercetin	Improves glycemic control Reduces lipid peroxidation Protects β-cells (by attenuating oxidative and inflammatory pathways)
Liu et al., 2014 [20]	EGCG	Regulates blood sugar Improves insulin sensitivity
Williams et al., 2004 [21]	Avicularin	Reduces oxidative stress by modulating crucial cellular pathways (PI3K; Akt/PKB; MAPK)
Agüero-Hernández et al., 2020 [24] Yang et al., 2022 [25] de-Araújo et al., 2018 [26] Palacz-Wrobel et al., 2017 [27]	Kaempferol	Regulates lipid metabolism Improves insulin resistance Enhances insulin signaling Restores the balance between glucose utilization and production Enhances antioxidant activity Influence inflammatory pathways (reduce TNF-α expression and secretion)
Palacz-Wrobel et al., 2017 [27]	Apigenin	Immune modulation and inflammatory response (reduce TNF-α expression and secretion; decreases IL-10 levels)
Huang et al., 2021 [28]	Bufadienolides	Influence apoptosis and autophagy

**Table 2:** The diverse range of effects of *K. pinnata* preparations in both in vivo and in vitro models of diabetes.

	Reference	Concentration/Doses	Observed Effects	Potential Benefits/Adverse Effects
1.	Halayal et al., 2024 [29]	In vitro: 0.0–40 µg/mL methanolic extract on 1% starch digestion with time.	Inhibition of alpha-amylase and alpha-glucosidase enzymes.	Potential therapeutic properties of *K. pinnata* in diabetes management.
2.	Singh et al., 2024 [30]	0.5% of *K. pinnata* extract administered to mice for 1 week.	Reduction in lipid levels.	Mitigation of hyperlipidemia and related metabolic disorders associated with diabetes.
3.	Menon et al., 2015 [31]	Aqueous *K. pinnata* leaves 0.14 g/Kg body weight administered to diabetic rats for 4 weeks.	Reduced body weight, blood glucose, cholesterol, IL-6, and increased BUN, IL-1β and TNF-α.	Management of glucose and cholesterol levels in diabetes.
4.	Matthew et al., 2013 [32]	Ethanolic and aqueous extracts of *K. pinnata* stem 300 & 600 mg/Kg body weight) were administered to diabetic rats for 12 days. In vitro: 0.063% & 0.125% ethanolic and aqueous extract on 1% starch digestion with time.	Decreased blood glucose levels occurred in a dose-dependent manner. α-amylase inhibition, but the ethanolic extract was more effective.	Anti-hyperglycemic activity.
5.	Ojewole, 2005 [33]	Aqueous leaf extract of *K. pinnata* was administered to diabetic rats at 25–800 mg/Kg body weight and monitored for 8 h.	Reduced blood glucose levels.	Hypoglycemic properties.
6.	Patil et al., 2013 [34]	Dichloromethane extract of *K. pinnata* leaves was administered to diabetic rats for 45 days. In vitro: rat pancreatic cells treated with 10 µg/mL of *K. pinnata* for 1 h.	Decreased glucose, glycated hemoglobin, lipid profiles & insulin levels. Increased insulin secretion com Parable to glibenclamide 10 µg/mL.	Effective in the therapy of diabetes.
7.	Ramon et al., 2023 [16] & Ramon et al., 2021 [35]	In vitro: Diabetic and non-diabetic skeletal muscle cells treated with combined *K. pinnata* and metformin preparations with combinatorial ratios varying from 5.0 mM metformin-only and 400 µg/mL of K. pinnata-only for 72 h.	Improved oxidative stress, downregulation of IL-6, and may promote inflammation.	May improve diabetes management and potential adverse effects in diabetes care. Immune-modulating activity that may promote beneficial or adverse effects.

## Data Availability

Data sharing is not applicable to this article.
